# Global prevalence of *Clostridioides difficile* in 17,148 food samples from 2009 to 2019: a systematic review and meta-analysis

**DOI:** 10.1186/s41043-023-00369-3

**Published:** 2023-04-18

**Authors:** Soroush Borji, Sepide Kadivarian, Shirin Dashtbin, Sara Kooti, Ramin Abiri, Hamid Motamedi, Jale Moradi, Mosayeb Rostamian, Amirhooshang Alvandi

**Affiliations:** 1 Department of Microbiology, School of Medicine, Kermanshah University of Medical Sciences, Kermanshah, Iran; 2 Department of Microbiology, School of Medicine, Iran University of Medical Sciences, Tehran, Iran; 3 Behbahan Faculty of Medical Sciences, Behbahan, Iran; 4 Fertility and Infertility Research Center, Research Institute for Health Technology, Kermanshah University of Medical Sciences, Kermanshah, Iran; 5 Infectious Diseases Research Center, Health Institute, Kermanshah University of Medical Sciences, Kermanshah, Postal Code: 6714415333 Iran; 6 Department of Microbiology, School of Medicine, Medical Technology Research Center, Research Institute for Health Technology, Kermanshah University of Medical Sciences, Kermanshah, Postal Code: 6714415333 Iran; 7 Student Research Committee, School of Medicine, Kermanshah University of Medical Sciences, Kermanshah, Iran

**Keywords:** *Clostridioides* (*Clostridium*) *difficile*, Food, Prevalence, Public health

## Abstract

**Background:**

*Clostridioides* (*Clostridium*) *difficile* is an important infectious pathogen, which causes mild-to-severe gastrointestinal infections by creating resistant spores and producing toxins. Spores contaminated foods might be one of the most significant transmission ways of *C. difficile*-associated infections. This systematic review and meta-analysis study were conducted to investigate the prevalence of *C. difficile* in food.

**Methods:**

Articles that published the prevalence of *C. difficile* in food in PubMed, Web of Science, and Scopus databases were retrieved using selected keywords between January 2009 and December 2019. Finally, 17,148 food samples from 60 studies from 20 countries were evaluated.

**Results:**

The overall prevalence of *C. difficile* in various foods was 6.3%. The highest and lowest levels of *C. difficile* contamination were detected to seafood (10.3%) and side dishes (0.8%), respectively. The prevalence of *C. difficile* was 4% in cooked food, 6.2% in cooked chicken and 10% in cooked seafood.

**Conclusions:**

There is still little known concerning the food-borne impact of C. difficile, but the reported contamination might pose a public health risk. Therefore, to improve the food safety and prevent contamination with *C. difficile* spores, it is necessary to observe hygienic issues during foods preparation, cooking and transfer.

**Supplementary Information:**

The online version contains supplementary material available at 10.1186/s41043-023-00369-3.

## Background

In the mid-1970s, the gram-positive and anaerobic bacterium *Clostridioides difficile* (formerly known as *Clostridium difficile*) was found as a common cause for nosocomial infection and a major cause of antibiotic-associated diarrhea [1–3]. By forming resistant spores and the ability of producing toxins, *C. difficile* is responsible for a diverse group of infection, from mild and self-limiting gastrointestinal infections to severe life threatening infections, like toxic megacolon [4, 5]. *C. difficile* infection (CDI) is associated with significant mortality and increased healthcare costs in the world [6–9]. *C. difficile* is basically a nosocomial pathogen, but the prevalence of community-acquired CDI seems to be increasing [10, 11]. Prevalence of *C. difficile* contamination in food is high, and a wide range of foods are contaminated by *C. difficile* [12, 13]. Therefore, consumption of *C. difficile* contaminated food is a risk factor for transmission of this infection in community, and one of the most important route of transmitting could be contaminated food by *C. difficile* spore [14, 15]. The presence of *C. difficile* in sewage-treatment plants might be a major reason of its community acquisition, transmission to food, and ultimately food contamination [14, 16]. This issue demands more attention to this health-threatening pathogen.

The main aims of this systematic and meta-analysis study were (i) to investigate the prevalence of *C. difficile* in different types of food and compare them with each other, (ii) to determine the frequency of toxin genes, (iii) to assay the relationship of toxin genes with the prevalence of *C. difficile*, and (iv) to evaluate the phenotypic and genotypic diagnostic methods from 17,148 food samples.

## Methods

### Literature search

Published studies from January 2009 to December 2019 were retrieved from four main databases including Web of sciences, Scopus, PubMed, and Google Scholar by applying the following keywords: “clostridia”, “*Clostridium* spp.”, “*Clostridium difficile*”, “*Clostridioides difficile*”, “*C. difficile*”, “antibiotic resistance”, “food contamination”, “toxinotype”, “ribotype”, and “toxin genes” alone or combined with ‘‘AND’’ and/or ‘‘OR’’ operators. To conduct the present study, Preferred Reporting Items for Systematic Reviews and Meta-Analysis (PRISMA) guideline were considered [17].

### Inclusion/exclusion criteria

All cross-sectional studies focusing on the prevalence of *C. difficile* contamination in food samples were included. Short communications, cohort studies, clinical trials, letter to editors, narrative or systematic reviews, and the non-English articles were excluded.

### Selection of studies and data gathering

The text of all included studies was accurately read by two independent authors, and in case of any discrepancy, the issue was discussed by other authors to be resolved. The following characteristics of each study were collected: first author, year of publication, sampling year, the location of the study, detection methods, sample type, sample size, the number of detected *C. difficile*, toxinotypes, ribotypes, toxin genes, antibiotics used, number of resistance isolates, and the method of antibiotic susceptibility assay.

### Data analysis

Data analyses were performed using Comprehensive Meta-Analysis software, V2.2.064. The *C. difficile* prevalence in different food samples and the prevalence of toxinotype and toxin genes, and antibiotic resistance rate in the *C. difficile* isolates were shown with event rate and a 95% confidence interval (CI). The random-effects model was chosen for meta-analyses, and several subgroup analyses were conducted to evaluate the source of heterogeneity based on the continent, country, sample types and the sampling periods of time. Using a random-effects model, risk ratios for each sample type were calculated to quantify the differences and rank the sample types based on the risk. The *Q* test and I2 statistic were applied to measure any possible heterogeneity between the studies. The publication bias was evaluated by conducting Egger weighted regression test. In all analyses, the significate threshold was < 0.05 (*p* value < 0.05).

## Results

### Search results

**Fig. 1:**
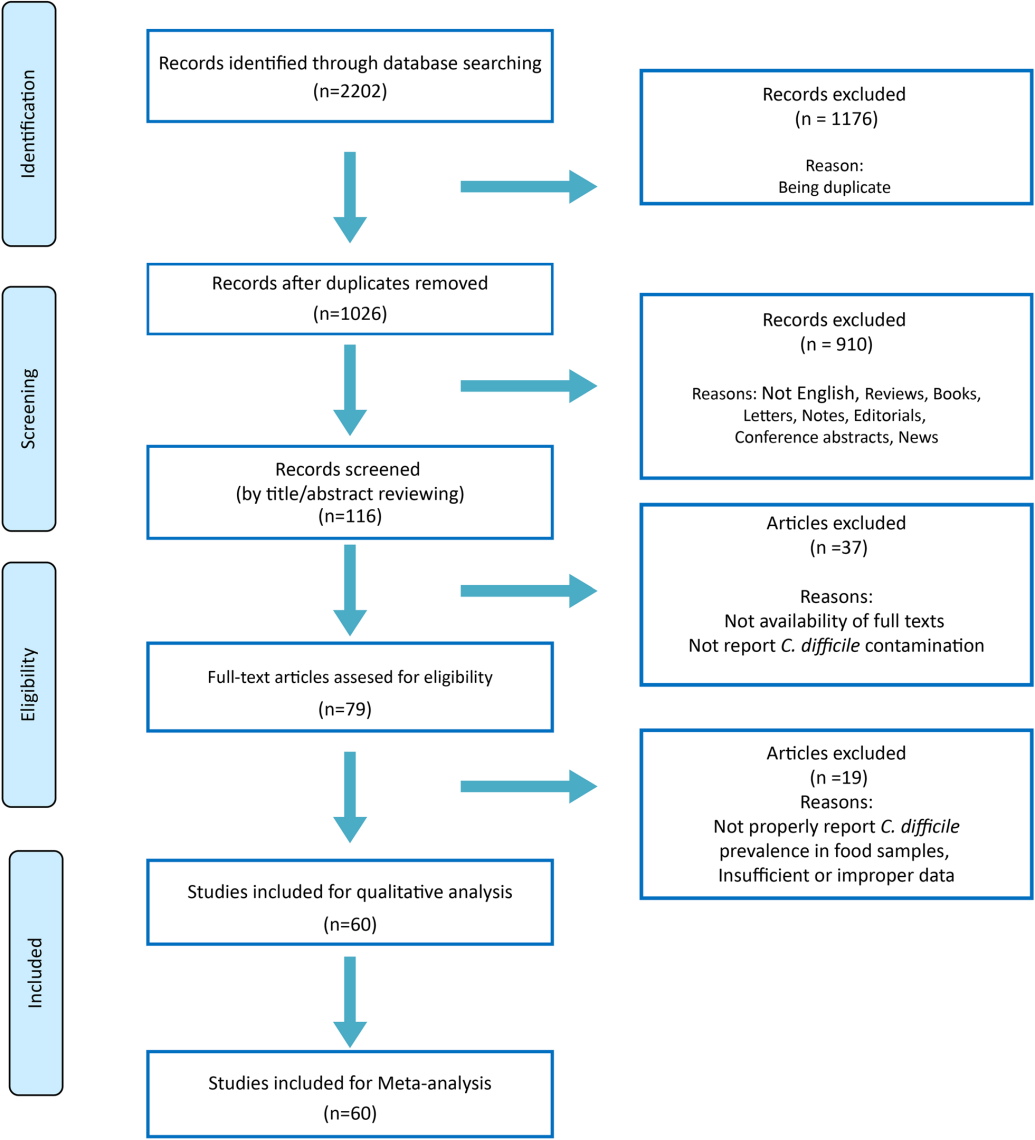
PRISMA flow diagram of study selection

In total, 2202 studies were recovered after accurate searching in the databases using the aforementioned key words. Among them, 1026 papers were non-duplicated articles and were considered in the study. After title/abstract screening, 116 studies remained. For eligibility, 79 studies were assessed by full-text reading. Sixty studies remained for final qualitative and meta-analysis. The diagram of our search strategy is given in Fig. 1, and the extracted characteristics of the studies are shown in Table 1.

**Table 1:** The characteristics of the studies

Study and Reference	Published year	Sampling year	Country	Continent	Detection method	Number of samples	Confirmed *C. difficile* No	Sample type	Sample size of each category	C.d isolates of each category	Antibiotic susceptibility assay	Antibiotic resistance*	Toxin genes*	Toxinotype*	Ribotype*
Abdel-Glil et al. [41]	2018	2014–2015	Egypt	Africa	PCR	150	0	R-meat	150	0	−	−	−	−	−
Kouassi et al. [42]	2014	2009–2010	Cote d'Ivoire		PCR	395	49	C-meat/Ham	395	49	DD	+	−	−	−
Esfandiari et al.-1 [43]	2014	2012	Iran	Asia	PCR	211	9	R-meat	166	9	−	−	+	−	+
								Soy	14	0					
								S-dishes	17	0					
								Veg	14	0					
Esfandiari et al.-2 [44]	–	2014	Iran		PCR	200	8	R-meat	200	8	−	−	+	−	+
Esfandiari- et al.-3 [45]	2013	2012–2013	Iran		−	211	22	R-meat	110	14	−	−	−	−	−
								Veg	70	8					
								Soy	14	0					
								S-dishes	17	0					
Esfandiari et al.-4 [46]	2014	−	Iran		PCR	100	12	R-meat	100	12	−	−	+	−	−
Hasanzade et al.-1 [47]	2013	−	Iran		−	240	25	R-poultry	240	25	−	−	−	−	−
Hasanzadeh et al.-2 [48]	2013	−	Iran		−	120	19	R-poultry	120	19	−	−	−	−	−
Kheradmand et al. [49]	2017	2014–2015	Iran		PCR	100	30	R-meat	100	30	−	−	+	−	−
Knight et al. [50]	2016	2013	Australia		PCR	300	76	R-meat	300	76	−	−	−	−	−
Kochakkhani et al. [51]	2017	2015	Iran		RT-PCR	60	8	Salad	60	8	DD	+	−	−	−
Lee et al. [52]	2018	2013–2014	South Korea		−	415	45	R-meat	266	20	E-test	+	+	−	−
								R-poultry	149	25					
Lim et al. [53]	2018	2015	Australia		PCR	300	30	Veg	300	30	−	−	+	−	+
Nayebpour et al.[54]	2018	2017	Iran		PCR	820	26	Seafood	820	26	DD	+	+	−	−
Rahimi et al.-1 [55]	2014	2012	Iran		PCR	660	13	R-meat	660	13	DD	+	+	−	+
Rahimi et al.-2 [56]	2015	2013	Iran		PCR	368	5	Salad	248	4	DD	+	+	−	−
								Milk/Dairy	50	1					
								S-dishes	70	0					
Rahimi et al.-3 [57]	2015	2012	Iran		PCR	570	6	C-meat/Ham	100	1	−	−	+	−	−
								R-meat	105	5					
								R-poultry	150	0					
								RTE meat	170	0					
Razmyar et al. [58]	2017	2014	Iran		PCR	65	10	R-poultry	65	10	−	−	+	−	−
Rivas et al. [59]	2019	2013–2014	New Zealand		RT-PCR	170	1	RTE meat	55	1	E-test	+	+	−	+
								Pet Food	6	0					
								Veg	6	0					
								R-meat	55	0					
								R-poultry	16	0					
								Milk/Dairy	2	0					
								Salad	27	0					
								Seafood	3	0					
Wu et al. [60]	2017	2015	Taiwan		PCR	302	67	R-meat	302	67	E-test	−	+	−	+
Yamoudy et al. [61]	2015	2013	Iran		PCR	106	6	Salad	106	6	−	−	+	−	−
Zamani et al. [62]	2019	2013–2014	Iran		PCR	30	2	R-meat	30	2	−	−	−	−	−
Curry et al. [63]	2012	2011–2012	USA	Central/North America	−	102	13	R-meat	102	13	−	−	+	−	+
Gomez et al. [64]	2013	2009–2010	Costa Rica		PCR	200	4	R-meat	133	3	E-test	+	+	−	+
								R-poultry	67	1					
Han et al. [65]	2018	2014–2015	USA		RT-PCR	297	41	Veg	297	41	MIC	+	+	−	+
Harvey et al.-1 [66]	2011	2004–2009	USA		PCR	243	20	R-meat	221	20	E-test	−	+	+	−
Harvey et al.-2 [67]	2011	2010	USA		PCR	32	7	R-poultry	32	7	E-test	+	+	+	−
Hawken et al.1 [68]	2013	–	Canada		PCR	80	8	R-meat	80	8	−	−	+	−	+
Hawken et al.-2 [69]	2013	–	Canada		PCR	78	4	R-meat	78	4	−	−	+	+	+
Houser et al. [70]	2010	–	USA		RT-PCR, PCR	80	0	R-meat	36	0	−	−	−	−	−
								R-poultry	17	0					
								Milk/Dairy	27	0					
Kalchayanand et al. [71]	2013	2006–2007	USA		PCR	2427	18	R-meat	2427	18	MIC	+	+	+	+
Kwon et al. [72]	2016	2011–2012	USA		PCR	910	2	S-dishes	380	1	−	−	+	−	+
								Veg	831	1					
								R-meat	308	0					
								R-poultry	142	0					
								Milk/Dairy	210	0					
Metcalf et al.-1 [73]	2010	2007–2008	Canada		PCR	393	7	R-meat	-	4	E-test	+	+	+	+
								C-meat/Ham	-	3					
Metcalf et al.-2 [74]	2011	2010	Canada		PCR	119	5	Seafood	28	4	E-test	+	+	+	+
								C-seafood	10	1					
Mooyottu et al. [75]	2015	–	USA		PCR	300	2	R-meat	200	2	E-test	+	+	−	−
								R-poultry	100	0					
Norman et al. [76]	2014	2012	USA		PCR	67	3	Seafood	67	3	E-test	+	+	+	−
Shaughnessy et al. [77]	2018	2011–2012	USA		PCR	342	0	R-meat	256	0	−	−	−	−	−
								R-poultry	109	0					
Songer et al. [28]	2009	2007	USA		PCR	88	37	R-meat	65	26	E-test	+	+	+	+
								RTE meat	23	11					
Varshney et al. [78]	2014	2011–2012	USA		RT-PCR, PCR	303	31	R-meat	150	14	E-test	+	+	−	+
								R-poultry	153	17					
Visser et al. [79]	2012	2007	Canada		PCR	48	3	R-meat	48	3	E-test	+	+	−	−
Weese et al.-1 [80]	2009	2008	Canada		PCR	230	28	R-meat	230	28	−	−	+	+	+
Weese et al.-2 [81]	2010	2008–2009	Canada		PCR	203	26	R-poultry	203	26	−	−	+	−	+
Agnoletti et al. [82]	2019	2015–2017	Italy	Europe	RT-PCR, PCR	702	113	Seafood	702	113	E-test	+	+	−	−
Abercron et al. [31]	2009	2008	Sweden		−	82	2	R-meat	65	2	−	−	+	−	−
								C-poultry	4	0					
								C-meat/Ham	13	0					
Bakri et al. [83]	2009	2008	UK		PCR	40	3	Veg	48	2	E-test	+	+	−	+
								Salad	24	1					
de Boer et al. [27]	2011	2008–2009	Netherland		RT-PCR, PCR	500	8	R-meat	243	1	−	−	+	−	+
								R-poultry	257	7					
Eckert et al. [84]	2013	2010–2011	France		PCR	104	3	Salad	60	2	DD	+	−	−	+
								Veg	44	1					
Ersoz et al. [29]	2018	2013–2014	Turkey		−	101	2	C-meat/Ham	25	2	E-test	+	−	−	−
								R-meat	31	0					
								R-poultry	27	0					
								C-poultry	12	0					
								RTE meat	6	0					
Guran et al. [85]	2015	2012–2013	Turkey		PCR	310	25	R-poultry	310	25	−	−	+	−	−
Hampikyan et al. [86]	2018	–	Turkey		PCR	555	161	R-meat	555	161	E-test	+	+	−	+
Jobstl et al. [87]	2010	2007–2008	Austria		PCR	120	3	R-meat	70	3	E-test	+	+	−	+
								Milk/Dairy	50	0					
Pasquale et al. [36]	2012	2010–2011	Italy		PCR	52	26	Seafood	52	26	−	−	+	+	+
Primavilla et al. [88]	2019	2016–2017	Italy		RT-PCR	350	2	C-meat/Ham	130	1	E-test	+	+	+	+
								Veg	54	1					
								S-dishes	166	0					
Rodriguez et al.-1 [89]	2014	2012	Belgium		PCR	240	8	R-meat	240	8	E-test, DD	+	+	−	+
Rodriguez et al.-2 [90]	2015	2013	Belgium		PCR	188	1	−	−	1	−	−	+	−	+
Rodriguez et al.-3 [24]	2013	2011–2012	Belgium		PCR	201	15	R-meat	201	15	−	−	+	−	+
Romano et al. [91]	2018	2009–2013	Italy		PCR	9	6	Milk/Dairy	6	6	−	−	+	−	+
								Salad	3	3					
Troian et al. [35]	2015	2012–2014	Italy		PCR	925	36	Seafood	925	36	E-test	+	+	−	+
Valerija et al. [20]	2018	2014–2017	Slovenia		PCR	154	28	Veg	154	28	−	−	−	+	+
Pires et al. [92]	2018	2017	Brazil	South America	RT-PCR	80	0	R-meat	80	0	−	−	−	−	−

*RT-PCR* Real-time PCR, *C-meat/Ham* Cooked meat/Hamburger, *Veg*. Vegetables, *S-dishes* Side dishes, *Seafood* Raw seafood/fish, *R-meat* Raw meat, *RTE meat* Ready-to-eat meat, *R-poultry* Poultry raw meat, *C-poultry* Cooked Poultry, *C-seafood* Cooked seafood/fish, *DD* Disk diffusion, *MIC* Minimum Inhibitory Concentration

^*^The columns show the presence (+) or absence (−) of the assigned parameters in the studies. More details of the parameters are represented in other tables or figures of the article

### The pooled prevalence of *C. difficile* in food samples

**Fig. 2:**
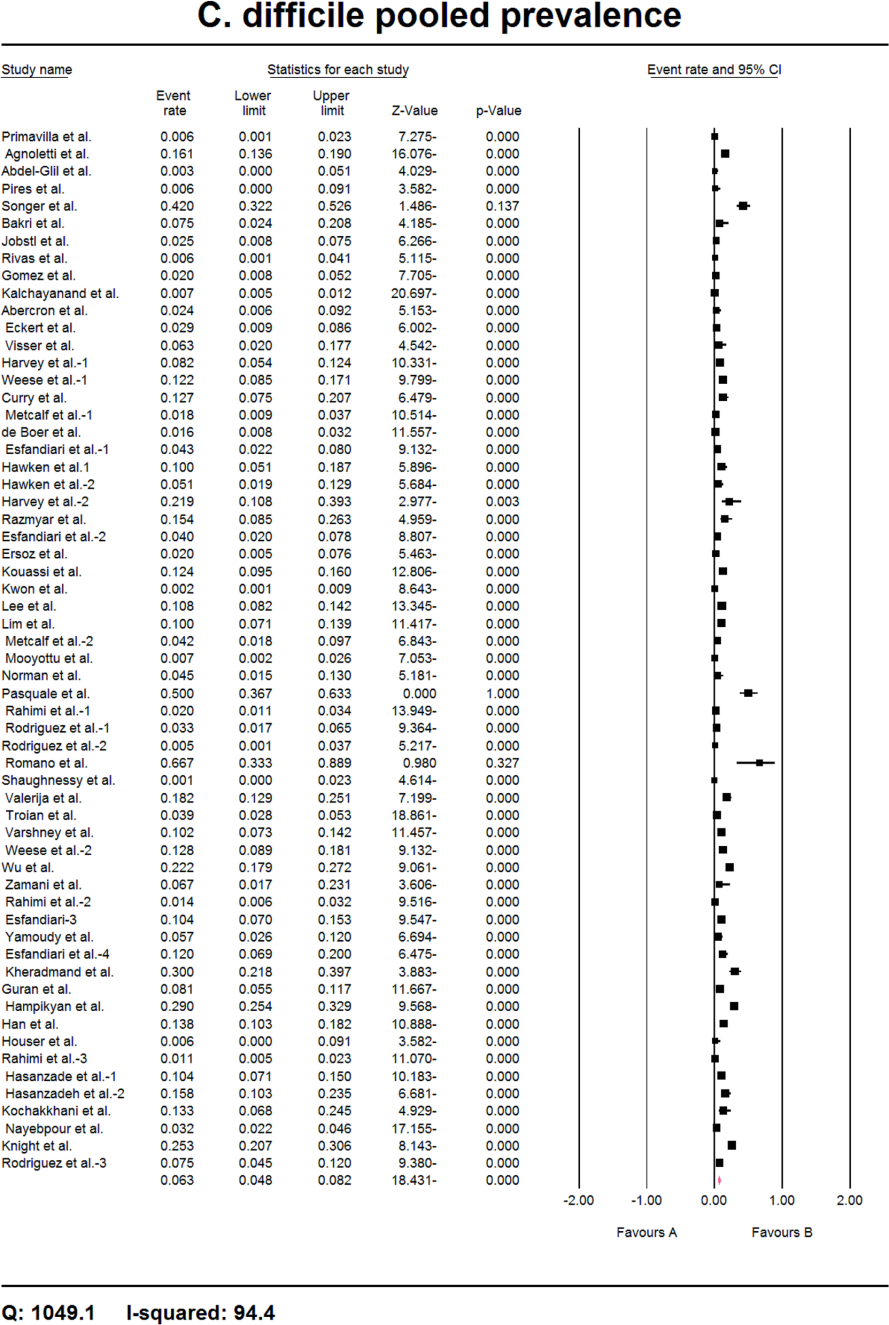
*C. difficile* pooled prevalence. The overall pooled prevalence of *C. difficile* in food samples was estimated to be 6.3% (CI 95%: 4.8–8.2). The lowest and highest *C. difficile* prevalence was observed in Shaughnessy et al. and Romano et al. reports with 0.1% and 66.7% prevalence, respectively

To analyze the pooled prevalence of *C. difficile* in food samples, 60 studies were used in a random-effects model. The event rate, which was the number of *C. difficile* cases over the number of samples, was applied as the effect size index. The overall pooled prevalence of *C. difficile* in food samples was estimated to be 6.3% (CI 95%: 4.8–8.2) (Fig. 2). The lowest and highest *C. difficile* prevalence was observed in Shaughnessy et al. and Romano et al. reports with 0.1% and 66.7% prevalence, respectively (Fig. 2). The *Q*-value was 1049.1 which was much higher than the number of studies minus 1 (60–1 = 59), that reject the null hypothesis and showed a significant heterogeneity between studies. The I^2^ statistics indicated that 94.4% of the variances reflect true variances between studies.

### Subgroup analysis of *C. difficile* prevalence based on the study continent, the year of sampling and the sample types

To subgroup analysis of *C. difficile* prevalence in food samples was performed based on the study continent, in which the 60 studies were divided into the following subgroups: Africa (two studies), Asia (20 studies), Central/North America (20 studies), Europe (17 studies), and South America (one study). Difference in prevalence of *C. difficile* isolated from food samples in different continents was not significant (Table 2).

**Table 2:** Subgroup analysis of *C. difficile* prevalence based on the studies continent, the sampling year and the sample types

Subgroup	Number of studies	Prevalence (%)	Lower limit	Upper limit	*Z*-value	*p* value	*I*^2^
*Based on the continent*							
Africa	2	4.8	0.8	24	− 3.2	0.001	85.6
Asia	20	7.4	4.6	11.7	− 9.9	0.000	93.8
Central/North America	20	5.1	3.1	8.4	− 10.9	0.000	94.1
Europe	17	6.8	3.9	11.3	− 9.1	0.000	95.6
South America	1	0.6	0.08	17.0	− 2.8	0.004	0.0
Overall	60	6.2	4.7	8.2	− 17.8	0.000	94.4
Test of heterogeneity between subgroups: *Q*-value:3.095, *p* value: 0.542							
*Based on the sampling year*							
TF1 (2004-the end of 2008)	8	5.1	2.3	10.9	− 6.9	0.000	97.0
TF2 (2009-the end of 2013)	23	5.7	3.6	9	− 11.2	0.000	93.6
TF3 (2014 ≤)	13	8.4	4.6	15.1	− 7.1	0.000	92.4
Overall	44	6.3	4.5	8.7	− 14.9	0.000	94.4
Test of heterogeneity between subgroups: *Q*-value: 1.372, *p* value: 0.504							
*Based on the sample types*							
C-meat/Ham	5	4.0	1.3	12.1	− 5.2	0.000	74.2
C-poultry	2	6.2	0.5	44.8	− 2.1	0.034	0.0
C-seafood	1	10.0	0.6	67.6	− 1.5	0.144	0.0
Milk/Dairy	6	4.5	1.2	15.7	− 4.3	0.000	77.6
Pet Food	1	7.1	0.2	72.8	− 1.4	0.157	0.0
R-meat	35	5.6	4.0	8.4	− 13.6	0.000	94.3
R-poultry	17	6.1	3.4	10.7	− 8.7	0.000	74.5
RTE meat	4	7.9	2.0	26.5	− 3.3	0.001	88.6
Salad	7	6.1	2.4	14.9	− 5.4	0.000	75.0
S-dishes	5	0.8	0.2	3.3	− 6.4	0.000	0.0
Seafood	7	10.3	4.6	21.4	− 4.9	0.000	96.4
Soy	2	3.3	0.3	29.1	− 2.7	0.008	0.0
Veg	10	5.7	2.6	11.8	− 6.9	0.000	77.7
Overall	102	5.7	4.5	7.3	− 21.6	0.000	90.2
Test of heterogeneity between subgroups: *Q*-value: 10.657, *p* value: 0.557							

To subgroup analysis of *C. difficile* food prevalence based on the sampling year, three time frames were used as follows: TF1 (2004-the end of 2008), TF2 (2009-the end of 2013), and TF3 (2014 ≤). Considering these time frames, 44 studies were used for a random-effects model subgroup analysis. No statistically significant difference was observed between time frame subgroups (Table 2).

To subgroup analysis of *C. difficile* food prevalence based on the sample type, the following subgroups were used: raw meat (R-meat), cooked meat/Hamburger (C-meat/Ham), poultry raw meat (R-poultry), cooked poultry (C-poultry), raw seafood/fish (Seafood), cooked seafood/fish (C-seafood), vegetables (Veg.), ready-to-eat meat (RTE meat), Milk/Dairy, salad, soy, side dishes (S-dishes), and pet food. The prevalence of *C. difficile* in each sample type is presented in Table 2. The highest and lowest prevalence were 10.3% and 0.8%, which were seen in Seafood and S-dishes sample types, respectively (Table 2). Although there were some differences in *C. difficile* prevalence of different sample types, no significant heterogeneity was observed between groups (*Q*-value: 10.657, *p* value: 0.557) (Table 2).

**Fig. 3:**
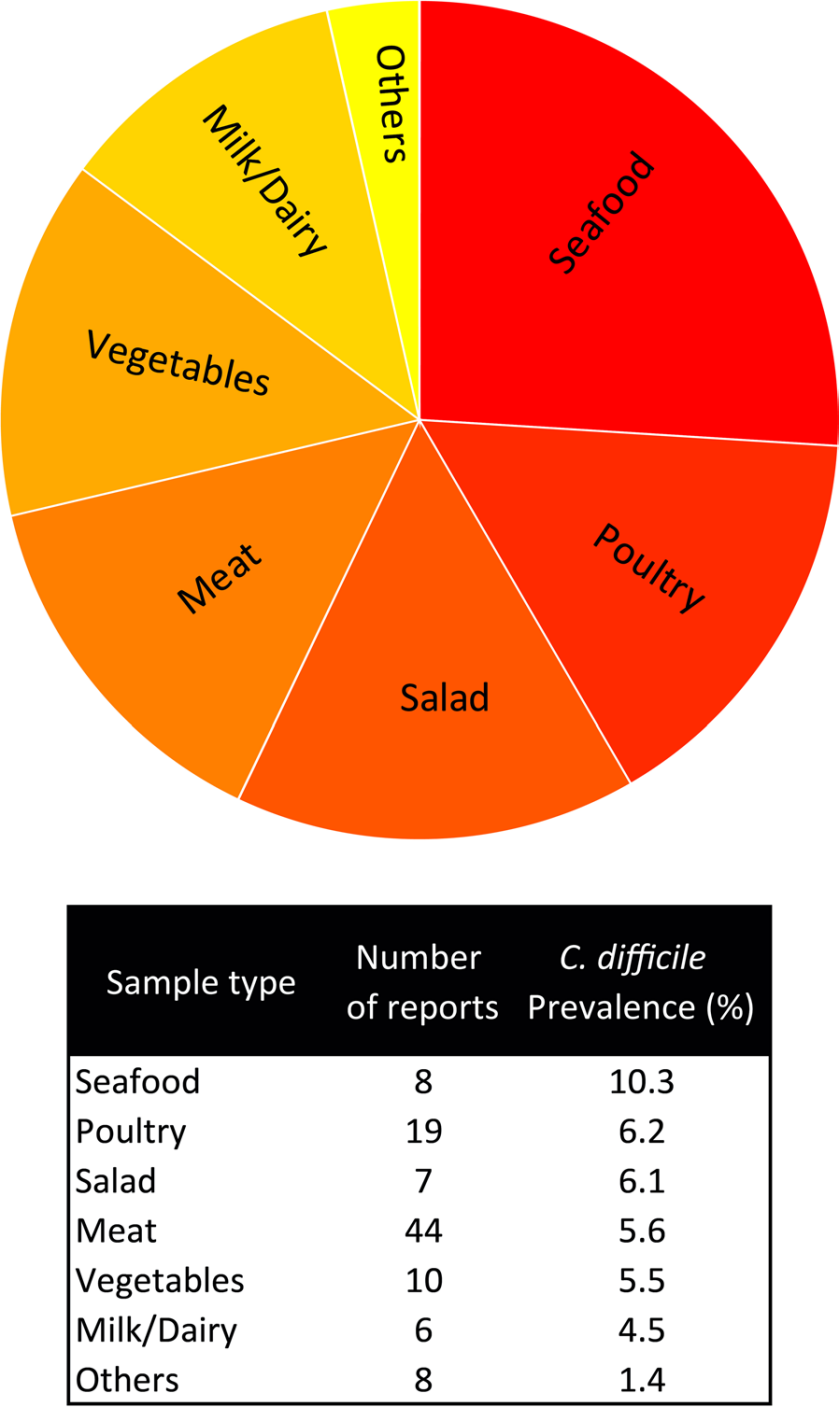
The prevalence of *C. difficile* in different sample types

For better presentation of the results, in another arrangement, the studies were divided to more general groups based on sample types as follows: meat, poultry, seafood, vegetables, salad, milk/diary, and others (S-dishes, soy, pet food) (Fig. 3).

**Fig. 4:**
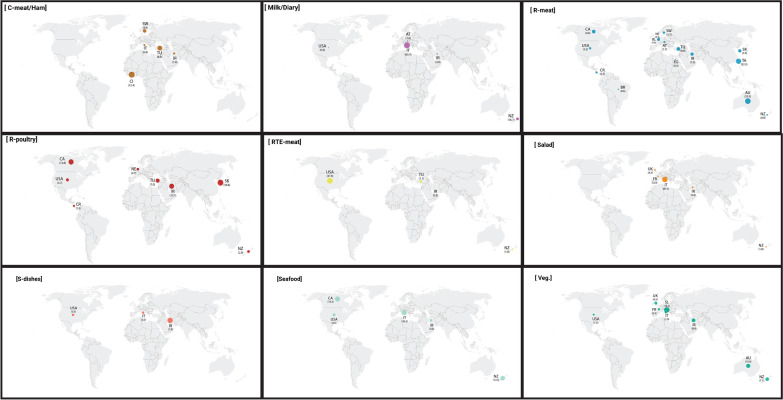
The prevalence of *C. difficile* in different sample types in each country. Each sample type is shown in a separate box. The overall prevalence of *C. difficile* in each country is presented with circles, and the real numbers of prevalence (in percentage) are also presented in parenthesis. *EG* Egypt, *CI* Cote d'Ivoire, *AT* Austria, *IR* Iran, *SK* South Korea, *TA* Taiwan, *NZ* New Zealand, *CA* Canada, *CR* Costa Rica, *USA* United States of America*,* *AT* Austria, *BL* Belgium, *FR* France, *IT* Italy, *NE* Netherland, Slovenia, *SW* Sweden, *TU* Turkey, *UK* United Kingdom, *BR* Brazil

**Fig. 5:**
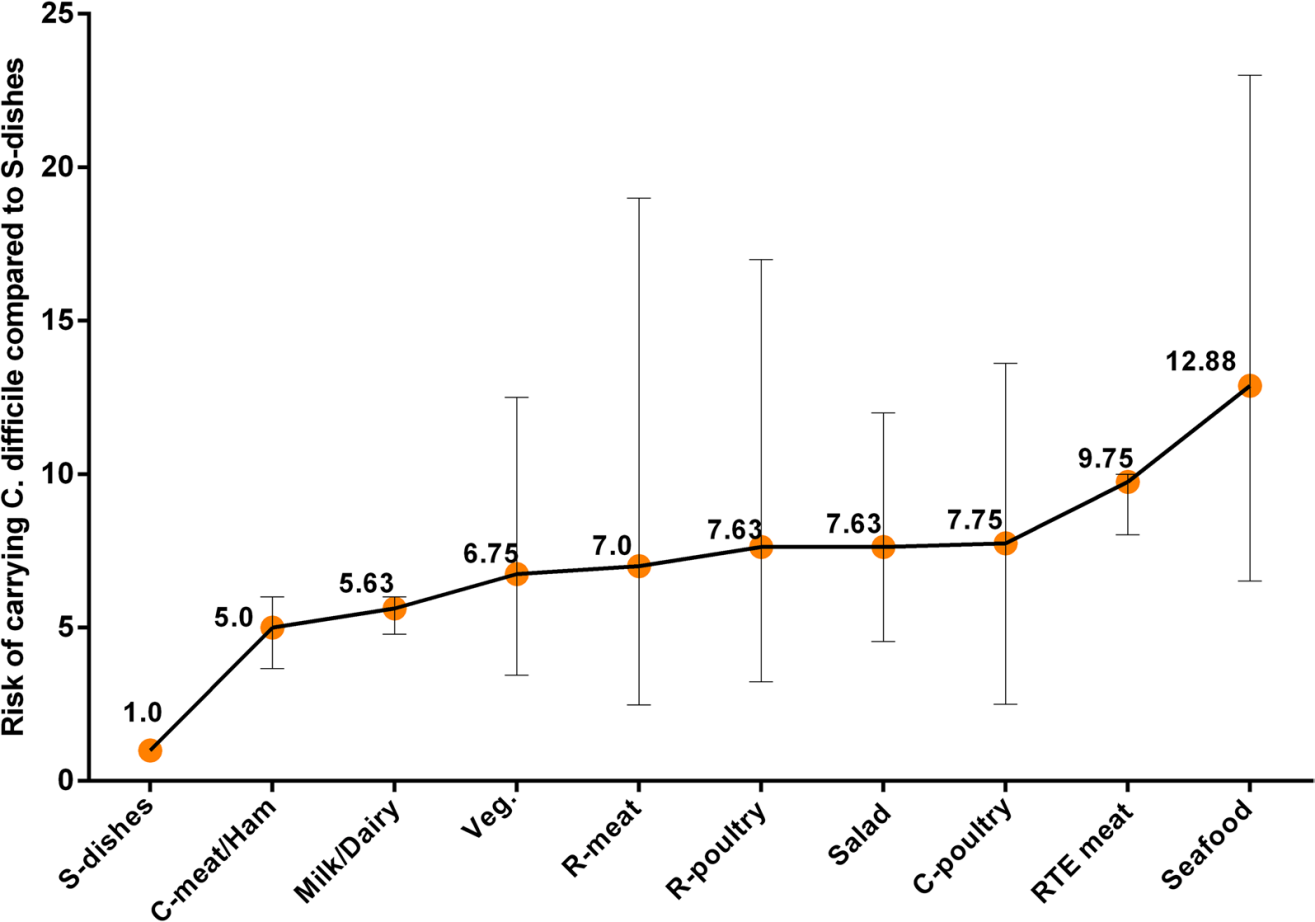
Ranking of *C. difficile* prevalence risk ratio per food type

For presenting each sample type in each country, more subgroup analyses were performed. The summary results of these analyses are shown in Fig. 4. Also, risk ratios were obtained using the extracted data. Based on the ranking of the risk ratio, S-dishes as a reference and was the lowest source of *C. difficile* and seafood, RTE meat, C-poultry, salad, R-poultry and R-meat had highest risks. Compared to S-dishes, the probability of contamination of seafood with CD was 12.88 times higher than S-dishes, and the risk of contamination of RTE meat, C-poultry, salad, R-poultry and R-meat obtained 9.75, 7.75, 7.63, 7.63 and 7.0 times more than S-dishes, respectively (Fig. 5).

### Prevalence of *C. difficile* ribotype, toxinotypes and toxin genes

According to a very diverse reported ribotypes, it was impossible to analyze the pooled prevalence of the ribotypes; this parameter is represented in Additional file 1: Table S1 without further analysis.

The most frequent toxinotypes of *C. difficile* were toxinotype 0, III, and V. As it is shown in Table 3, the toxinotype V was more prevalent comparing to other two toxinotypes, and there was a significant heterogeneity between the toxinotypes (*Q*-value: 9.725, *p* value: 0.008) (Table 3).

**Table 3:** The prevalence of *C. difficile* toxinotypes and toxin genes

Toxinotype	Number of studies	Prevalence (%)	Lower limit	Upper limit	*Z*-value	*p* value	*I*^2^
*C. difficile* toxinotypes							
0	4	15.6	4.5	42.2	− 2.4	0.016	71.9
III	5	22.7	8.3	48.8	− 2.0	0.041	58.4
V	9	64.5	41.9	82.1	1.3	0.205	79.2
Overall	18	39.1	25.2	54.9	− 1.4	0.175	79.9
Test of heterogeneity between subgroups: *Q* value: 9.725, *p* value: 0.008							

Toxin genes	Number of studies	Prevalence (%)	Lower limit	Upper limit	*Z*-value	*p* value	*I*^2^
*C. difficile* toxin genes detected by molecular methods							
A	37	76.8	68.1	83.8	5.3	0.000	64.6
B	37	75.9	66.7	83.2	5.0	0.000	68.7
cdtA	3	28.8	10.1	59.2	− 1.4	0.165	0.0
CTD	27	49.6	36.9	62.3	− 0.1	0.953	79.0
tcdC	6	41.7	20.6	66.3	− 0.6	0.516	72.6
tcdC117	4	17.6	5.3	44.7	− 2.3	0.023	63.5
tcdC18	9	19.5	9.6	35.5	− 3.4	0.001	0.0
tcdC39	9	67.4	48.9	81.7	1.9	0.064	68.7
Overall	132	61.7	56.2	67.0	4.1	0.000	74.7
Test of heterogeneity between subgroups: *Q*-value: 58.9, *p* value: 0.000							

The toxin genes that were reported in more than one study include genes A, B, CTD, tcdC, tcdC18, tcdC39, tcdC117, and cdtA. The toxin genes of A and B were the most frequent, and genes tcdC18 and tcdC117 were the lowest frequent genes studied (Table 3). There was also significant heterogeneity between the studied genes (*Q*-value: 58.9, *p* value: 0.000) (Table 3).

As shown in Table 4, toxin type 0 in which pathogenic strains were located shows a higher prevalence in seafood samples. While the prevalence of toxin types 3 and 5 was higher in RTE meat and R-poultry. As shown in Table 5, the highest prevalence of toxin genes A, B, and CDT was observed in RTE meat samples. Compared to other samples, Milk/Dairy and Salad rank after RTE meat in terms of the high prevalence of genes A and B toxins.’

**Table 4:** The prevalence of *C. difficile* toxinotypes in each sample type

Toxinotype	Sample type	Number of studies	Prevalence (%)	Lower limit	Upper limit	*Z*-value	*p* value	*I*^2^
O	R-meat	3	8.81	3.32	21.35	− 4.43	0.00	0.000
	Seafood	1	42.31	25.20	61.49	− 0.78	0.43	0.000
	Overall	4	26.05	15.91	39.60	− 3.291	0.001	71.943
Test of heterogeneity between subgroups: *Q*-value: 9.433, *p* value: 0.002								
III	R-meat	4	19.93	7.55	43.11	− 2.45	0.01	64.128
	RTE meat	1	36.36	14.33	66.12	− 0.89	0.37	0.000
	Overall	5	26.58	13.69	45.24	− 2.413	0.016	58.436
Test of heterogeneity between subgroups: *Q*-value: 0.966, *p* value: 0.326								
V	R-meat	5	75.65	49.87	90.66	1.95	0.05	75.058
	R-poultry	1	93.75	46.14	99.62	1.85	0.06	0.000
	RTE meat	1	63.64	33.88	85.67	0.89	0.37	0.000
	Seafood	2	38.03	3.87	90.33	− 0.35	0.73	79.274
	Overall	9	70.95	53.09	84.05	2.275	0.023	78.361
Test of heterogeneity between subgroups: *Q*-value: 2.99, *p* value: 0.393

**Table 5:** The prevalence of *C. difficile* toxin genes in each sample type

Toxin gene	Sample type	Number of studies	Prevalence (%)	Lower limit	Upper limit	*Z*-value	*p* value	*I*^2^
A	Milk/Dairy	1	92.86	42.28	99.57	1.75	0.08	0.00
	R-meat	21	82.84	70.03	90.89	4.25	0.00	72.93
	R-poultry	4	66.43	44.94	82.75	1.51	0.13	28.75
	RTE meat	1	95.83	57.54	99.74	2.17	0.03	0.00
	Salad	2	87.50	46.27	98.27	1.82	0.07	0.00
	Seafood	6	59.70	50.15	68.56	1.99	0.05	28.90
	Veg	2	79.71	57.76	91.86	2.54	0.01	13.03
	Overall	37	68.76	62.02	74.79	5.180	0.000	64.57
Test of heterogeneity between subgroups: *Q*-value: 15.01, *p* value: 0.020								
B	Milk/Dairy	1	92.86	42.28	99.57	1.75	0.08	0.00
	R-meat	21	81.53	66.35	90.80	3.61	0.00	75.38
	R-poultry	4	61.19	40.70	78.36	1.07	0.28	26.09
	RTE meat	1	95.83	57.54	99.74	2.17	0.03	0.00
	Salad	2	87.50	46.27	98.27	1.82	0.07	0.00
	Seafood	6	56.78	43.42	69.22	0.99	0.32	57.52
	Veg	2	95.61	58.20	99.71	2.20	0.03	43.95
	Overall	37	68.38	59.75	75.91	4.016	0.000	68.68
Test of heterogeneity between subgroups: *Q*-value: 14.96, *p* value: 0.020								
CDT	Milk/Dairy	1	16.67	2.28	63.13	− 1.47	0.14	0.00
	R-meat	14	58.10	35.39	77.83	0.69	0.49	70.99
	R-poultry	4	49.23	11.15	88.22	− 0.03	0.98	78.35
	RTE meat	1	95.83	57.54	99.74	2.17	0.03	0.00
	Salad	2	87.50	46.27	98.27	1.82	0.07	0.00
	Seafood	5	28.58	11.54	55.12	− 1.60	0.11	79.94
	Overall	27	51.16	36.47	65.66	0.152	0.879	79.02
Test of heterogeneity between subgroups: *Q*-value: 13.20, *p* value: 0.021								
tcdC18	R-meat	7	17.29	10.83	26.45	− 5.65	0.00	0.00
	R-poultry	1	23.53	9.12	48.55	− 2.06	0.04	0.00
	RTE meat	1	36.36	14.33	66.12	− 0.89	0.37	0.00
	Overall	9	20.35	13.96	28.68	− 5.893	0.000	0.00
Test of heterogeneity between subgroups: *Q*-value: 2.28, *p* value: 0.32								
tcdC39	R-meat	6	63.98	39.88	82.63	1.14	0.25	78.03
	R-poultry	1	76.47	51.45	90.88	2.06	0.04	0.00
	RTE meat	1	63.64	33.88	85.67	0.89	0.37	0.00
	Seafood	1	91.67	37.82	99.50	1.62	0.10	0.00
	Overall	9	69.81	55.46	81.12	2.654	0.008	68.67
Test of heterogeneity between subgroups: *Q*-value: 1.94, *p *value: 0.58								
tcdC	R-meat	4	42.31	9.65	83.43	− 0.32	0.75	74.84
	R-poultry	1	70.00	37.63	90.02	1.23	0.22	0.00
	Seafood	1	23.08	10.75	42.76	− 2.59	0.01	0.00
	Overall	6	37.08	22.57	54.37	− 1.472	0.141	72.61
Test of heterogeneity between subgroups: *Q*-value: 6.13, *p* value: 0.05								
tcdC117	R-meat	4	16.70	4.15	48.15	− 2.05	0.04	63.48
	Overall	4	16.70	4.15	48.15	− 2.054	0.040	63.48
Test of heterogeneity between subgroups: *Q*-value: 0.00, *p* value:1.0								
cdtA	R-meat	2	27.28	12.80	48.95	− 2.05	0.04	0.00
	Seafood	1	30.77	16.20	50.55	− 1.91	0.06	0.00
	Overall	3	29.20	18.12	43.47	− 2.786	0.005	0.00
Test of heterogeneity between subgroups: *Q*-value: 0.07, *p* value: 0.79								

### Publication bias

The publication bias was checked based on the pooled prevalence of *C. difficile* isolates in food samples. The Egger’s linear regression test result showed a significant publication bias in the included studies (*p* value < 0.0001).

## Discussion

Consuming the contaminated raw and cooked foods with *C. difficile* spore might be an important route of its transmission [18–20]. Food contamination has played an important role in epidemiology of some infectious diseases, but little information is available about the global frequency of *C. difficile* in food products [21, 22]. The present study analyzed the distribution of *C. difficile* in 60 studies published from 2009 to 2019 in 17,148 food samples. The results showed that the overall prevalence of *C. difficile* in all food samples was 6.3%, with the lowest and highest prevalence of *C. difficile* were 0.1% and 66.7%, respectively. In a systematic review study, Rodriguez-Palacios and colleagues reported the 4.1% prevalence of *C. difficile* in human diet samples during 1981 to 2018 [21]. Comparing to the results presented in this study, it seems that the reported prevalence of the bacterium in these two studies is quiet the same. Taken together, the overall *C. difficile* prevalence in food samples in the world seems to be less than 10%, but it is relatively high and should not be undermined.

Significant heterogeneity was observed between the studies that indicated different prevalence of *C. difficile* in different parts of the world. However, in addition to real differences in *C. difficile* prevalence, the observed heterogeneity may be due to different seasons of sampling, temperatures and geographical conditions, the quality of studies, the sensitivity of detection methods, etc. [23]. Although the frequency of *C. difficile* varied in food samples from different continents, these differences were not statistically significant. The prevalence of *C. difficile* in Asia and Europe was almost the same, but it was lower in Africa and North/Central America comparing to similar reports [21]. In this study, this difference could be attributed to high consumption of seafood’s in diet of Asia and Europe, and a large number of seafood samples have been studied. The lowest prevalence of *C. difficile* was observed in South America.

Most of the studies were on meat and meat products. The contamination of undercooked and prepared foods was evident [24]. The prevalence of *C. difficile* in meat products of this study was the same as a report by Usui 2020 [25], but was lower than study reported from Canada by Warriner in 2017 [26]. It must be noted that the prevalence of food sample isolated *C. difficile* was so variable with a range of 1.6% from Netherlands [27] to 42% from USA [28]. The prevalence of *C. difficile* in chicken and poultry meat was 6.2%, which was similar to the previous study (6.7%) [25]. However, the isolation rate of *C. difficile* from chicken meat samples was ranging from %0 [29–31] to 44.4% in turkey meat samples [32]. It seems that the chicken with skin is more vulnerable to contamination comparing to skin-less chicken samples [33].

Seafood and oysters well-known carriers of *C. difficile* [34]*.* In the present meta-analysis, the overall contamination rate of seafood was 10.3% and had the highest risk ratio (12.88%). According to another meta-analysis study, pooled prevalence of *C. difficile* in seafood was shown a little bit more in comparison with our pooled prevalence (Seafood risk ratio was 14.3) [21]. This difference may be because of longer time and more included studies. The variation between prevalence of *C. difficile* isolated from seafood’s have been seen in many studies from around the world ranged from 3.9% to more than 40% [35–37]. The first report of root vegetables contamination with *C. difficile* was in 1996 [38].

In this study, the overall prevalence of *C. difficile* in contaminated vegetables was 5.7%, which was less than another meta-analysis (12% on average). This would be due to the increase of health level in production and transfer of vegetables [25, 26].

Regardless of the type of food products, the most important issue in relation to *C. difficile* strains is detecting their ribotypes and toxinotypes [24]. Although we could not statistically analyze the *C. difficile* ribotypes data due to vast divergence of the informations, it is obvious that ribotypes 027 and 078 were the most predominants followed by 001 [20, 39], 010 [33] and 020/014 [20] ribotypes. The results of the present study showed that the most common toxinotypes were toxinotypes were toxinotype V, 0, III, respectively. In a review study, the presence of toxin genes in food samples was estimated as 3.5% [32] to 100% [31, 32, 37]. The types of toxinotypes can be important in the development of molecular diagnostic tests and vaccines [40].

As reported by many studies *C. difficile* harboring *tcd*A and *tcd*B, toxin genes were more prevalent than other strains [31].

The contamination risk analysis showed that seafood and RTE-meat are the high-risk foods. While in Rodriguez-Palacios study (21), among different food items, vegetables and seafood were ranked as the high-risk food items, in both studies, seafood is one of the risk food items. This information can be useful for determining preventive food safety measures (cooking food and not consuming raw food) to minimize the possibility of further food contamination.

This study showed that a variety of foods, especially seafood, were at potential risk for *C. difficile*. The frequency of *C. difficile* varied in food samples from different continents. This difference can be attributed to the high consumption of seafood in the diet of Asia and Europe. These results suggest that consumption of raw and undercooked foods is a way to further transmit *C. difficile* to humans.

## Conclusions

Therefore, enough cooking of food, suitable washing of animal carcasses in the slaughter process, prevention of carcass contamination with animal feces play an important role in increasing food safety.

## Supplementary Information

**Additional file 1.** The ribotypes of the studies.

## Data Availability

All relevant data are within the manuscript and its Supporting Information files.
